# Unexpected Actors in Inflammatory Bowel Disease Revealed by Machine Learning from Whole-Blood Transcriptomic Data

**DOI:** 10.3390/genes13091570

**Published:** 2022-09-01

**Authors:** Jan K. Nowak, Cyntia J. Szymańska, Aleksandra Glapa-Nowak, Rémi Duclaux-Loras, Emilia Dybska, Jerzy Ostrowski, Jarosław Walkowiak, Alex T. Adams

**Affiliations:** 1 Department of Pediatric Gastroenterology and Metabolic Diseases, Poznan University of Medical Sciences, 60572 Poznan, Poland; 2 INSERM U1111, Centre International de Recherche en Infectiologie, Université Claude Bernard Lyon 1, 69364 Lyon, France; 3 Department of Genetics, Maria Skłodowska-Curie National Research Institute of Oncology, 02781 Warsaw, Poland; 4 Department of Gastroenterology, Hepatology and Clinical Oncology, Centre for Postgraduate Medical Education, 01813 Warsaw, Poland; 5 Translational Gastroenterology Unit, Nuffield Department of Medicine, Experimental Medicine Division, University of Oxford, John Radcliffe Hospital, Oxford OX3 9DU, UK

**Keywords:** inflammatory bowel disease, Crohn’s disease, ulcerative colitis, expression, TGF-beta

## Abstract

Although big data from transcriptomic analyses have helped transform our understanding of inflammatory bowel disease (IBD), they remain underexploited. We hypothesized that the application of machine learning using lasso regression to transcriptomic data from IBD patients and controls can help identify previously overlooked genes. Transcriptomic data provided by Ostrowski et al. (ENA PRJEB28822) were subjected to a two-stage process of feature selection to discriminate between IBD and controls. First, a principal component analysis was used for dimensionality reduction. Second, the least absolute shrinkage and selection operator (lasso) regression was employed to identify genes potentially involved in the pathobiology of IBD. The study included data from 294 participants: 100 with ulcerative colitis (48 adults and 52 children), 99 with Crohn’s disease (45 adults and 54 children), and 95 controls (46 adults and 49 children). IBD patients presented a wide range of disease severity. Lasso regression preceded by principal component analysis successfully selected interesting features in the IBD transcriptomic data and yielded 12 models. The models achieved high discriminatory value (range of the area under the receiver operating characteristic curve 0.61–0.95) and identified over 100 genes as potentially associated with IBD. *PURA*, *GALNT14*, and *FCGR1A* were the most consistently selected, highlighting the role of the cell cycle, glycosylation, and immunoglobulin binding. Several known IBD-related genes were among the results. The results included genes involved in the TGF-beta pathway, expressed in NK cells, and they were enriched in ontology terms related to immunity. Future IBD research should emphasize the TGF-beta pathway, immunoglobulins, NK cells, and the role of glycosylation.

## 1. Introduction

Inflammatory bowel diseases (IBDs) present a major societal challenge as they remain debilitating and incurable conditions. Their molecular dissection at multiple levels has allowed for the identification of key genes involved in intestinal and immune homeostasis. Several strategies have proven fit for this purpose: the search for monogenic causes of very early-onset IBD [1,2], genome-wide association studies (GWAS) [3,4], and experiments in animal models of gut inflammation [5].

Our growing knowledge of the genetic architecture of IBD points toward the crucial interactions with the environment via regulatory mechanisms. Two promising approaches include transcriptome and methylome analysis. The former has aided in recognizing the genes behind signals is GWAS [6] and has shown the capacity for discovering druggable targets [7]. It seems, however, that the potential of transcriptomics in uncovering hidden IBD pathobiology remains underexploited as the development of pragmatic expression-powered applications gains steam.

The rise of machine learning (ML) approaches presents a new opportunity for using existing transcriptomic data in the study of IBD, as applied in some other fields [8]. These chances come with challenges, two of which should be mentioned. Firstly, the results processed by artificial intelligence are often difficult to decipher, consisting of large datasets themselves. Secondly, ML algorithms are considered a “black box” whose function may depend on factors of which the medical researcher is unaware. These problems can be addressed by least absolute shrinkage and selection operator (lasso) regression by performing feature selection to provide only the most relevant results. Lasso regression also works in a way that is possible to retrace. We hypothesize that the application of ML using lasso regression applied to transcriptomic data from IBD patients and controls can help identify previously overlooked genes—the unexpected actors in IBD [9].

## 2. Materials and Methods

This study was built from a large dataset provided by Ostrowski et al. who searched for blood transcriptome biomarkers in patients with ulcerative colitis (UC), Crohn’s disease (CD), and controls [10]. This Polish multicenter study was designed to enroll approximately 50 participants in each of these categories, including adult and pediatric subjects, for a total of approximately 300 participants. The IBD groups were heterogenous, with disease activity ranging from remission (0) to an active flare (4 according to Ostrowski et al.). Briefly, blood was collected in Tempus tubes, analyzed using the Ion AmpliSeq panel on an Ion Proton (all Thermo Fisher), mapped to hg19 using the Torrent Mapping Alignment Program, and counted using *htseq-count* v. 0.6 [11]. The methods used by Ostrowski et al. in their study were described in detail by members of this team in an earlier publication [12]. The raw data are accessible from the European Nucleotide Archive (PRJEB28822). 

### Statistical Analysis

The analyses were performed using the R language v. 3.6.2 (R Foundation for Statistical Computing, Vienna, Austria). We removed genes with an expression ≤10, as well as sample duplicates, and we filtered the data for quality to obtain transcriptome data (13,264 genes) from 294 study participants. Because of the complete availability of data, which allowed for an independent quality check, our dataset slightly differs from the original set employed by Ostrowski et al. (the exact selection of patients was not available in the data). The counts table was subjected to further normalization using *DESeq2* v. 1.24.0, the addition of one (the offset value), and log_2_ transformation [13]. This allowed the modification of the read distribution prior to ML and the preservation of the initial zero values as null. 

The following classification problems were investigated in all study participants, as well as in adults and children separately: IBD vs. control, UC vs. control, and CD vs. control. Furthermore, models were built for differentiating patients with active disease vs. grouped controls and patients in remission: overall, in UC, and in CD. For each explored contrast, an adequate subset (e.g., data from UC patients) underwent principal component analysis (PCA) with scaling using the *feature_select_PCA* function from *clustifyr* v. 1.6.0 [14]. Top transcripts explaining 95% of the variance in the first three principal components were selected for further analysis. The aim was to preselect variables most representative of small-scale clusters of co-expression, which—crucially—also resulted in a reduction in collinearity. Binomial lasso regression was conducted using the *glmnet* package [15]; the α parameter was set at 1 and the values of λ ranged from 0.01 to 0.4 (by 0.01). Importantly, the upper (0.1) and lower (−0.1) limits were set for coefficients. Model performance was measured using the area under the receiver-operating characteristic curve (AUC) for the classification of IBD and control cases. Following tenfold cross-validation, the median AUC value was calculated for the models with non-null discriminative values (AUC > 0.5) to select the optimal lambda value. Therefore, the final model was selected on the basis of a tradeoff between model precision and the strength of shrinkage. Moreover, setting the aforementioned limits on coefficient values ensured that a few of the most highly contributing genes entered the models. The cross-validation process provided not only estimates of performance but also results; the number of occurrences of each transcript in 10 models across all lambda values was counted and summarized. Gene set enrichment analysis (GSEA) was conducted using the following GSEA collections: gene ontology (C5) and canonical pathways (CP), which included Reactome, KEGG, BioCarta, and the Pathway Interaction Database [16]. The GSEA web interface available at www.gsea-msigdb.org was used (The Broad Institute, Cambridge, MA, USA). A dataset by Peters et al. containing both blood and intestinal tissues from CD patients was employed to explore the potential correlations of interesting genes (R2 platform, University of Amsterdam, Amsterdam, the Netherlands) [17].

## 3. Results

The study included data from 294 participants: 100 with UC (48 adults and 52 children), 99 with CD (45 adults and 54 children), and 95 controls (46 adults and 49 children). The age range was 1–69 years. The majority of participants (57%) were female, and this trend persisted in all subgroups. The disease was active in seven adults with UC (15%), seven adults with CD (16%), 31 pediatric patients with UC (60%), and 29 pediatric patients with CD (54%). 5-Aminosalicylates were used in approximately 90% of IBD patients, immunosuppressants were used in 20% of UC patients and 50% of CD patients, and steroids were used in 50% of pediatric CD patients and 25% of all adults with IBD. Biologics were administered to 30% of adult CD and pediatric UC patients and to 12% of adult UC and pediatric CD participants (please see Table 1 in the original study by Ostrowski et al. [10]).

Nine models were built to compare IBD, UC, and CD patients to controls, including those with active and mild disease, as well as separate analyses for adults and children. Full results can be found in Supplementary Table S1. The value of the models measured using AUC ranged from 0.61 (mild IBD vs. controls) to 0.95 (IBD in children vs. controls). The smallest number of included genes was two (severe CD vs. controls) and the highest was 27 (severe IBD vs. controls).

The study identified over 100 genes potentially associated with IBD. Table 1 presents the results of the analyses that included the largest number of patients. All three models achieved considerable discriminatory power (AUC 0.83–0.87). However, the shrinkage coefficient was relatively low in the analysis of IBD vs. controls, which led to the inclusion of a large number of transcripts in the final model. One of the most consistent associations with IBD was *SYTL2* (synaptotagmin-like 2), and the association was negative. Interestingly, in the data from Peters et al., *SYTL2* was negatively correlated with the IBD-related transcripts *SPI1* (Spi-1 proto-oncogene; *r* = −0.95, *p* = 3.42 × 10^−279^), *NFE2* (nuclear factor, erythroid 2; *r* = −0.95, *p* = 6.11 × 10^−268^), and *TGFB1* (transforming growth factor beta 1; *r* = −0.95, *p* = 4.02 × 10^−267^). Other interesting results included the proinflammatory mediator *CYSLTR1* (cysteinyl leukotriene receptor 1; negative association with IBD) and *BLVRB* (biliverdin reductase B; greater in IBD), which not only converts biliverdin to bilirubin but also acts on riboflavin that may improve CD symptoms [18]. Interestingly, *BLVRB* was positively associated with *TGFB1* (*r* = 0.87, *p* = 9.8 × 10^−161^) and negatively correlated with *ITGAV* (integrin subunit alpha V; *r* = −0.89, *p* = 3.3 × 10^−181^). ITGAV takes part in the latent activation of TGF-beta, as well as in the activation of SMAD5 (SMAD family member 5; *r* = −0.88, *p* = 2.0 × 10^−173^), which is more easily phosphorylated when TGF-beta levels are low. This suggests a negative feedback loop from high levels of TGF-beta. Although *PGF* (placental growth factor; positively associated with IBD) did not have a strong correlation, *TGFBR2* was one of the 10 most prominent ones (transforming growth factor beta receptor 2; *r* = 0.68, *p* = 4.6 × 10^−72^). *GALNT14* (polypeptide *N*-acetylgalactosaminyltransferase 14) was also included in the model; it takes part in protein glycosylation, a process shown to be involved in IBD [19]. Overall, despite lasso regression selecting many transcripts with unknown functions and significances (*NPSR1-AS1* and *YBX3P1*), there seems to be an underlying biological theme related to the TGF-beta pathway and factors known to be important from other studies: *SPI1* (PU.1) and *NFE2* [20].

Interestingly, severe IBD (Table 2) was related to a gene that we previously showed to be associated with the need for treatment escalation in UC (in the long term), independent of CRP: *CACNA1E*, calcium voltage-gated channel subunit alpha 1 E [21]. The aforementioned glycosylating protein *GALNT14* was included in the analysis of active IBD, along with *ITGB4* (integrin subunit beta 4), which plays a role in IGF-1 signaling (insulin-like growth factor 1) and was linked to a case of UC [22]. The antibody receptors *FCGR1A* and *FCGR1B* (Fc gamma receptors Ia and Ib) were also found in the results, along with Toll-like receptor *TLR5* and cluster of differentiation *CD274*, which is better known as PD-L1 (programmed death ligand 1), an immunosuppressive protein targeted by oncological therapies. Of note, *CACNA1E*, *GALNT14*, *ITGB4*, and *FCGR1A* were considered to be potential biomarkers in the original study conducted by the members of this team when the data were analyzed using a standard approach. Lasso regression provided additional results that warrant attention, e.g., JCHAIN (joining chain of multimeric IgA And IgM). Thus, a key theme of antibody production, antibody binding, and immune regulation can be readily identified in these results.

The accuracy of the IBD recognition model in children was the highest, allowing for a greater shrinkage of coefficients (Table 3). These results included *TAS2R31*, a taste receptor that binds saccharin (which may alter gut microbiota and was proposed to have a role in IBD etiology) and acesulfame K, both of which are artificial sweeteners. Farnesyltransferase *FNTA* (farnesyltransferase, CAAX box, alpha) was also among the results, along with *BLK* tyrosine kinase (BLK proto-oncogene, Src family tyrosine kinase), which may phosphorylate FCGR2A, -B, and -C, and which is correlated with IBD genes *ZAP70* (zeta chain of T-cell receptor-associated protein kinase 70; *r* = 0.81, *p* = 1.4 × 10^−121^) and *ITGAL* (integrin subunit alpha L; *r* = 0.80, *p* = 4.1 × 10^−119^). In adults, the main results involved the vesicle-related *AP3S1* (adaptor related protein complex 3 subunit sigma 1) that correlated with *ITGAV* (*r* = 0.88, *p* = 2.5 × 10^−167^) and *TGFB1* (*r* = −0.87, *p* = 2.4 × 10^−161^), which seems analogous to *BLVRB* (albeit inverse). Killer cell lectin *KLRF1* (killer cell lectin-like receptor F1) was included, which is expressed chiefly by natural killer (NK) cells. *PURA* (purine-rich element-binding protein A) correlates positively with *TGFBRAP1* (transforming growth factor beta receptor-associated protein 1; *r* = 0.79, *p* = 4.79 × 10^−112^). Overall, the most frequently selected genes (Table 1, Table 2 and Table 3) included *NPSR1-AS1*, *PIK3C2A*, *PURA*, *TAS2R31*, and *YBX3P1* (all *n* = 4), followed by *FCGR1A*, *GALNT14*, *SNORA19*, *SP140L*, and *STIM2* (*n* = 3). From the cross-validation data, the most consistently selected genes included *GALNT14*, *PURA*, and *FCGR1A*.

**Figure 1:**
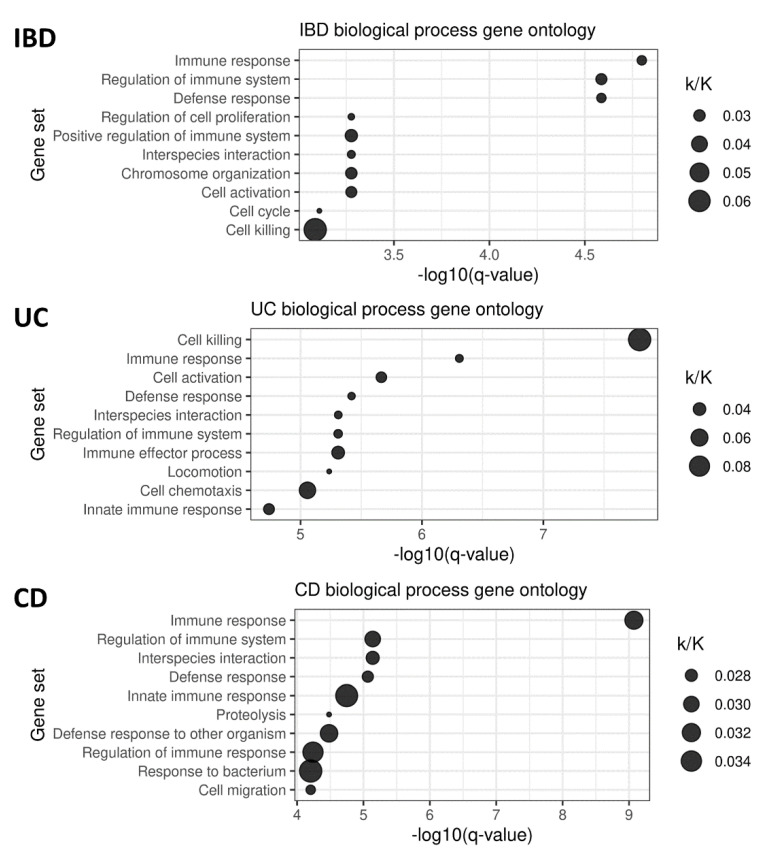
Gene ontology analysis of the genes included in the lasso models for the prediction of IBD, UC, and CD status. The ratio of the number of genes in overlap (k) to all genes in the given gene set (K) is indicated.

The GSEA of transcripts selected by the lasso regression models within the cross-validation loop revealed the enrichment of processes typically associated with IBD, immune responses, and cell activation (Figure 1). UC ontology strongly pointed toward cellular killing mechanisms. Apart from the immune system, in general, CD was associated with innate immunity, proteolysis, and the host’s response to bacteria.

## 4. Discussion

We applied an ML algorithm to IBD transcriptomic data and obtained a list of genes that may be involved in the pathogenesis of IBD. At study conception, the source expression data from Ostrowski et al. presented the richest whole-blood IBD transcriptome and had not been explored using ML methods. The results from lasso regression implicate inflammation, the TGF-beta pathway, immunoglobulins, NK cells, and other mechanisms in the pathogenesis of IBD.

Interestingly, most of the genes identified by PCA and lasso were not among the top differentially expressed genes in the original study by Ostrowski et al. [10]. This can potentially be explained by less pronounced, more systematic differences. In children, individuals that expressed the most differential transcripts in Ostrowski et al. had very high AUC values within the test set, even superior to those obtained by lasso. However, the diagnostic accuracy was found to be lower in an independent replication using qPCR. In adults, the highest AUC achieved by Ostrowski et al. in the validation cohort using a set of five genes was 0.78 (active UC). Our study, using a set of two genes, was capable of identifying active CD with an average AUC of 0.92 in 10-fold cross-validation. However, it is uncertain how this classifier would perform when translated to qPCR. These results suggest that the approach by Ostrowski et al. was highly efficient. Consequently, in this study, we did not propose any diagnostic panels.

Lasso provides an alternative approach for the analysis of transcriptomic data and is capable of drawing attention to new targets. A lasso-derived classifier is not a grouping of all the genes that strongly differ between two groups (many of which are correlated), but a selection of complementary transcripts which can hypothetically represent various co-expression clusters and pathways. For this reason, we did not utilize final models as the input for GSEA but rather focused on the list of genes obtained from cross-validation. The results appear to be typical for IBD biology and were strongly related to immune system activation and control, as well as cell activation and survival. The employed gene ontology was rich and contained thousands of gene sets. Nevertheless, the most enriched functional categories were broad. The biological themes identified by the investigation of the final lasso models (presented in the tables) are more specific.

Firstly, the results bring attention to the TGF-beta pathway, which is disrupted in IBD. Defects in TGF-beta processing and signaling (e.g., *ITGAV*), especially in dendritic cells, can trigger colitis [23]. The microbiota likely impacts TGF-beta signaling within the intestinal mucosa to promote renewal and regeneration. The genes selected for the models do not belong to the TGF-beta pathway but display several striking correlations, as in the case of *SYTL2* and *BLVRB*. It can be inferred that, in this study, IBD was positively associated with TGF-beta production but negatively associated with its processing. In this cross-sectional transcriptomic analysis, it is difficult to distinguish between the cause (core IBD characteristics) and effects (inflammation), as well as the mechanisms meant to contain inflammatory damage.

Secondly, attention is focused on the processes involving immunoglobulins. Anti-commensal IgG antibodies are present in the mucosa of patients with UC and lead to the induction of interleukin-1β through Fc gamma receptors [24]. *FCGR2A*, a gene indicated by the GWAS of IBD, is involved in this process. *FCGR1A* and *FCGR1B* (the expression of which tends to correlate) may play a role in IBD as demonstrated by their association with a lack of response to anti-tumor necrosis factor α (TNF) [25]. In the current study, *FCGR1A* had a strong positive correlation with active CD. Yet, *BLK*—which phosphorylates *FCGR2A*—was negatively associated with an IBD diagnosis. Again, identifying the nature of the dynamic interactions between these actors using transcriptomics is challenging and calls for mechanistic studies.

Thirdly, the results confirm the link between IBD and NK cells. *SYTL2*, the most consistent result in the comparison between IBD and controls, was recently shown to strongly associate with NK cell markers in the peripheral blood [26]. It is also one of the most frequently mutated genes in colorectal cancer [27], the risk of which is increased in IBD. *SYTL2* was suggested to be related to MAP kinases, which is of interest because, in this study, *DUSP3* was overexpressed in patients with active CD, possibly testifying to the negative control of the kinases. Returning to NK cells, two other genes were also negatively associated with IBD within the lasso models: *KIR2DL1* (killer cell immunoglobulin-like receptor, two Ig domains and long cytoplasmic tail 1) and *KLRF1*. Killer cell immunoglobulin genes were also linked to IBD by genetic studies, including *KIR3DL1*, *KIR3DL2* [28], *KIR2DL2*, and *KIR2DL1*. *KLRF1* encodes a C-type lectin receptor (which binds CLEC2B, also known as AICL, to activate TNF production). We recently showed that one of the C-type lectins (*CLEC5A*) strongly correlates with treatment escalation in UC [20]. Additionally, evidence regarding the importance of C-type lectins/receptors in IBD is accumulating. Of note, these genes can be expressed not only in NK cells, but also in specific subsets of T cells (e.g., naïve activated CD4^+^ cells, i.e., receptors) and monocytes/macrophages (ligands). There seems to be a clear negative association between NK cell-related transcript abundance and IBD, and it is of great interest as to which cell subtypes are responsible for the observed effects.

Lastly, several other results put the spotlight on various aspects of IBD inflammation. GALNT14 plays a role in glycosylation, and its association with the immune system can be inferred from its usefulness as a predictor in several cancers. *TLR5* (Toll-like receptor 5) knockout mice may serve as a model of colitis that is dependent on the presence of microbiota [29]. The aforementioned *CD274* (PD-L1) is widely known for its key role in the immunosuppressive tumor microenvironment and is a target for cancer therapies (together with PD-L1 receptor, PD-1), which can cause colitis through the activation of resident memory CD8^+^ T cells [30]. There is more exciting IBD biology to be explored, including ion channels (*CACNA1E*) and leukotrienes (*CYSLTR1*). Genes of unknown significance that were consistently selected by the models include *PURA* (in UC), which plays a role in the cell cycle and may cause myelodysplasia in case of hemizygosity. Even though the complexity of inflammation draws attention to a large number of fascinating genes, this knowledge alone may be insufficient to uncover the origin of the disease and to pinpoint the persisting changes in the organism that are responsible for recurrences in IBD.

The presence of multiple transcripts that are strongly associated with IBD confirms that our choice of methods was correct. Yet, there are limitations to the standard two-stage feature selection process that included PCA and lasso regression. Firstly, a majority of transcripts were excluded as they did not explain the variance within the dataset sufficiently well. Secondly, lasso may prefer one variable over many that are correlated despite minute differences between them. Therefore, the results may be unstable even when differences between datasets are minimal. For this reason, a large number of models were built, and only the most consistent results were discussed and accompanied by pertinent transcriptomic correlates. This is why we attached a Supplementary Table S1 with a list of the most consistent results from the models built within the cross-validation loop. The use of lasso enabled our study to avoid overfitting and to select key results. Because the original study design by Ostrowski et al. benefits from subgroup stratification, statistical compensation for confounding variables, such as gender and age, was unnecessary.

## 5. Conclusions

In conclusion, the application of ML to IBD transcriptomic data obtained by Ostrowski et al. allowed us to identify several plausible targets for future mechanistic investigation. In particular, the obtained results highlight the role of the TGF-beta pathway, immunoglobulins, and NK cells.

## Figures and Tables

**Table 1:** Genes were selected by lasso regression to best discriminate patients with IBD from controls; both adults and children were included. Coefficients are presented together with the number of times the given transcript appeared in all tested model across the cross-validation/lambda grid (greater values indicate transcripts more systematically linked to IBD). The top three genes (highest *n*) are indicated in bold. The area under the curve (AUC; 90% confidence interval), lambda shrinkage parameter, and the intercept for each model are also presented. Please note that the genes with the most discriminatory power are present at the top and the bottom of the list.

IBD		Ulcerative Colitis		Crohn’s Disease	
AUC = 0.85 (0.79–0.92), λ = 0.11		AUC = 0.87 (0.79–0.95), λ = 0.16		AUC = 0.83 (0.75–0.83), λ = 0.14	
Gene	Coefficient, *n*	Gene	Coefficient, *n*	Gene	Coefficient, *n*
*(Intercept)*	0.79	*(Intercept)*	0.06	***PGF***	**0.05, 218**
*SERINC2*	0.05, 122	*TTC14*	−0.01, 38	***YWHAG***	**0.05, 164**
*GALNT14*	0.05, 112	*RBBP6*	−0.01, 8	***THEM5***	**0.05, 153**
*BLVRB*	0.05, 105	*TAS2R31*	−0.01, 44	*(Intercept)*	0.04
*PGF*	0.03, 110	*YBX3P1*	−0.02, 146	*EMC3*	0.02, 104
*TK1*	0.02, 89	*TOP2B*	−0.05, 50	*DUSP3*	0.00, 19
*BCAM*	0.01, 108	***STIM2***	**−0.05, 195**	*PIK3C2A*	−0.02, 44
*MSMP*	−0.03, 66	*CHML*	−0.05, 159	*MSMP*	−0.05, 140
*LINC00938*	−0.04, 44	***PURA***	**−0.05, 297**	*PARP15*	−0.05, 95
*UBR7*	−0.04, 93	*CYSLTR1*	−0.05, 153	*ANKRD36*	−0.05, 91
*TAS2R31*	−0.04, 30	*ARAP2*	−0.05, 150	*PEG10*	−0.05, 112
*SP140L*	−0.05, 30	*SP140L*	−0.05, 129	*KIR2DL1*	−0.05, 94
*FLJ40194*	−0.05, 92	*STXBP4*	−0.05, 164	*ERMARD*	−0.05, 101
*STIM2*	−0.05, 105	***SNORA19***	**−0.05, 233**	*YBX3P1*	−0.05, 141
*PIK3C2A*	−0.05, 135	*NPSR1−AS1*	−0.05, 138	*PAXIP1−AS1*	−0.05, 69
*CYSLTR1*	−0.05, 129	*LINC00938*	−0.05, 44		
*SMG6*	**−0.05, 142**	*FLJ40194*	−0.05, 161		
*RTTN*	−0.05, 127				
*SYTL2*	**−0.05, 160**				
*NPSR1-AS1*	−0.05, 125				
*YBX3P1*	**−0.05, 141**				

**Table 2:** Genes were selected by lasso regression to best discriminate patients with severe IBD from controls. Coefficients are presented together with the number of times the given transcript appeared in the tested models across the cross-validation/lambda grid (greater values indicate transcripts more systematically linked to IBD). The top three genes (highest *n*) are indicated in bold. The area under the curve (AUC, 90% confidence interval), lambda shrinkage parameter, and the intercept for each model are also presented. Please note that genes with the most discriminatory power are present at the top and the bottom of the list.

Severe IBD		Severe Ulcerative Colitis		Severe Crohn’s Disease	
AUC = 0.91 (0.83–0.98), λ = 0.13		AUC = 0.90 (0.73–1.0), λ = 0.15		AUC = 0.92 (0.79–1.0), λ = 0.25	
Gene	Coefficient, *n*	Gene	Coefficient, *n*	Gene	Coefficient, *n*
*ITGB4*	0.05, 171	*ITGB4*	0.05, 196	***FCGR1A***	**0.05, 270**
***FCGR1A***	**0.05, 275**	***BIK***	**0.05, 234**	***DUSP3***	**0.05, 141**
*FCGR1B*	0.05, 116	*CACNA1E*	0.05, 193	*(Intercept)*	−0.97
*SEMA4A*	0.05, 60	*PGLYRP1*	0.05, 138		
***CACNA1E***	**0.05, 219**	*DOK4*	0.05, 131		
*PLP2*	0.05, 93	*LRRC61*	0.05, 122		
*TK1*	0.05, 143	***GALNT14***	**0.05, 251**		
*TLR5*	0.05, 67	*TXNDC5*	0.05, 129		
*CD274*	0.05, 119	*JCHAIN*	0.05, 152		
*OPLAH*	0.05, 77	*NATD1*	0.05, 88		
*DOK4*	0.05, 137	*FCGR1A*	0.03, 117		
***GALNT14***	**0.05, 300**	*NQO2*	0.02, 84		
*JCHAIN*	0.05, 216	*IGLL5*	0.01, 96		
*THEM5*	0.05, 124	*SMARCAD1*	−0.01, 63		
*IGLL5*	0.01, 96	*LYPD2*	−0.03, 73		
*GPR160*	0.00, 44	*PIK3C2A*	−0.05, 108		
*RAD23A*	0.00, 93	***PURA***	**−0.05, 265**		
*TAS2R31*	0.00, 47	*SNORA19*	−0.05, 183		
*DDX12P*	−0.03, 122	*NPSR1−AS1*	−0.05, 185		
*SNORA19*	−0.03, 55	*(Intercept)*	−0.97		
*CRYGS*	−0.04, 93				
*PURA*	−0.05, 54				
*LYPD2*	−0.05, 95				
*SCARNA5*	−0.05, 123				
*NPSR1-AS1*	−0.05, 205				
*YBX3P1*	−0.05, 124				
*LOC200772*	−0.05, 142				
*(Intercept)*	−0.27				

**Table 3:** Genes were selected by lasso regression to best identify IBD in children and adults (adult patients presented a more quiescent disease relative to children). Coefficients are presented together with the number of times the given transcript appeared in all tested model across the cross-validation/lambda grid (greater values indicate transcripts more systematically linked to IBD). The top three genes (highest *n*) are indicated in bold. The area under the curve (AUC; 90% confidence interval), lambda shrinkage parameter, and the intercept for each model are also presented. Please note that the genes with the most discriminatory power are present at the top and the bottom of the list.

IBD Children		IBD Adults	
AUC = 0.95 (0.89–1.0), λ = 0.20		AUC = 0.86 (0.75–0.98), λ = 0.15	
Gene	Coefficient, *n*	Gene	Coefficient, *n*
*(Intercept)*	0.78	*(Intercept)*	0.73
*NBEAL1*	0.05, 132	*ADAMTS1*	0.00, 118
***FNTA***	**0.05, 234**	*METTL14*	−0.01, 45
*RAD23A*	0.05, 178	*STIM2*	−0.03, 31
*BLVRB*	0.02, 137	*SLX4IP*	−0.05, 142
*BCAM*	0.01, 176	*MYBL1*	−0.05, 63
*BIK*	0.00, 154	***DTHD1***	**−0.05, 164**
***BLK***	**−0.05, 201**	***AP3S1***	**−0.05, 254**
***TAS2R31***	**−0.05, 237**	*PIK3C2A*	−0.05, 156
*PAXIP1-AS1*	−0.05, 41	*PURA*	−0.05, 174
		***KLRF1***	**−0.05, 197**
		*ERMARD*	−0.05, 59
		*SP140L*	−0.05, 83
		*SYTL2*	−0.05, 96

## Data Availability

The data are publicly available from the original study: European Nucleotide Archive accession number PRJEB28822.

## Supplementary Materials

The following supporting information can be downloaded at https://www.mdpi.com/article/10.3390/genes13091570/s1: Table S1. Complete results.
